# A case report of acute intermittent porphyria leading to severe disability

**DOI:** 10.3389/fneur.2023.1334743

**Published:** 2024-01-11

**Authors:** Jie Lin, Jinzhi Liu, Aihua Wang, Zhihua Si

**Affiliations:** ^1^Department of Neurology, Shandong Provincial Qianfoshan Hospital, Shandong University, Shandong Institute of Neuroimmunology, Shandong Key Laboratory of Rheumatic Disease and Translational Medicine, Shandong, China; ^2^Department of Neurology, The First Affiliated Hospital of Shandong First Medical University & Shandong Provincial Qianfoshan Hospital, Shandong Institute of Neuroimmunology, Shandong Key Laboratory of Rheumatic Disease and Translational Medicine, Shandong, China

**Keywords:** acute intermittent porphyria, abdominal pain, limb weakness, epilepsy, psychiatric abnormalities

## Abstract

Acute intermittent porphyria (AIP) is a rare inherited metabolic disorder resulting from increased production of porphyrins and their precursors, δ-aminolevulinic acid (ALA) and porphobilinogen (PBG), due to deficiencies in the enzymatic activity of the heme synthesis pathway. The disease is typically characterized by a triad of abdominal pain, neurologic impairment symptoms, and psychiatric abnormalities. However, only a small percentage of patients present with this classic triad of symptoms. Our female patient, aged 23, was admitted to the hospital with a 4-year history of abnormal mood episodes and weakness in the limbs for over 1 week. She had a previous medical history of intestinal obstruction. After admission, a cranial MRI revealed reversible posterior leukoencephalopathy imaging manifestations, and the patient exhibited weakness of the extremities, respiratory failure, seizures, and severely reduced serum sodium concentration. The diagnosis of AIP was ultimately confirmed by a positive urine PBG-sunlight test and analysis of HMBS gene variants. The absence of typical triadic signs in acute attacks of AIP can make early recognition of the disease challenging. We present a case with multiple typical clinical manifestations of AIP in the hope of aiding clinicians in fully recognizing acute intermittent porphyria.

## Introduction

Porphyrias are a group of metabolic disorders resulting from impaired heme biosynthesis, leading to abnormally high concentrations of porphyrins or their precursors that accumulate in tissues and cause cellular damage. These diseases are primarily caused by enzyme defects and are inherited in an autosomal dominant manner with low epistasis (1). They can be categorized into two groups based on the site of porphyrin production: erythropoietic porphyrias, characterized by skin damage and minimal neurological involvement, and hepatic porphyrias, characterized by neurological and visceral signs and symptoms (2). Hepatic porphyrias is subdivided into acute hepatic porphyria and chronic hepatic porphyria. Acute hepatic porphyrias (AHPs) is a complex group of inborn errors of metabolism that result in acute episodic neurovisceral attacks, encompassing four life-threatening disorders—acute intermittent porphyria (AIP), hereditary coproporphyria (HCP), variegate porphyria (VP), and delta- or 5-aminolevulinic acid (ALA) dehydratase deficiency (or Doss porphyria) (ALADP). The mode of inheritance of acute hepatic porphyria is autosomal dominant with low penetrance, except for ALAD-deficiency hematoporphyria (ALADP), which is autosomal recessive. AIP is classified as a subtype of acute hepatic porphyria and is the most frequently encountered type of this disorder (3, 4). AIP is a hereditary disease resulting from defects in the HMBS gene encoding porphobilinogen deaminase (PBG deaminase), the third key enzyme in the heme synthesis pathway. Deficiency of this enzyme leads to the accumulation of toxic porphyrin precursors, primarily δ-aminolevulinic acid (ALA) and porphobilinogen (PBG). Due to its toxic impact on nerve fibers, AIP can manifest clinically as abdominal pain, neuropathy, and psychiatric abnormalities (5). AIP is a rare disease often overlooked by clinicians. As patients may not exhibit multiple typical symptoms simultaneously, there can be a considerable delay between the first symptom and the diagnosis of porphyria, resulting in decreased quality of life, permanent neuropathy, severe and long-lasting sequelae, and even death. Here, we present a case of acute intermittent porphyria with an acute onset of severe limb weakness progressing to respiratory muscle weakness and respiratory failure. This case report aims to raise awareness among clinicians and facilitate early recognition and accurate diagnosis of this rare disease.

## Case

**Figure 1:**
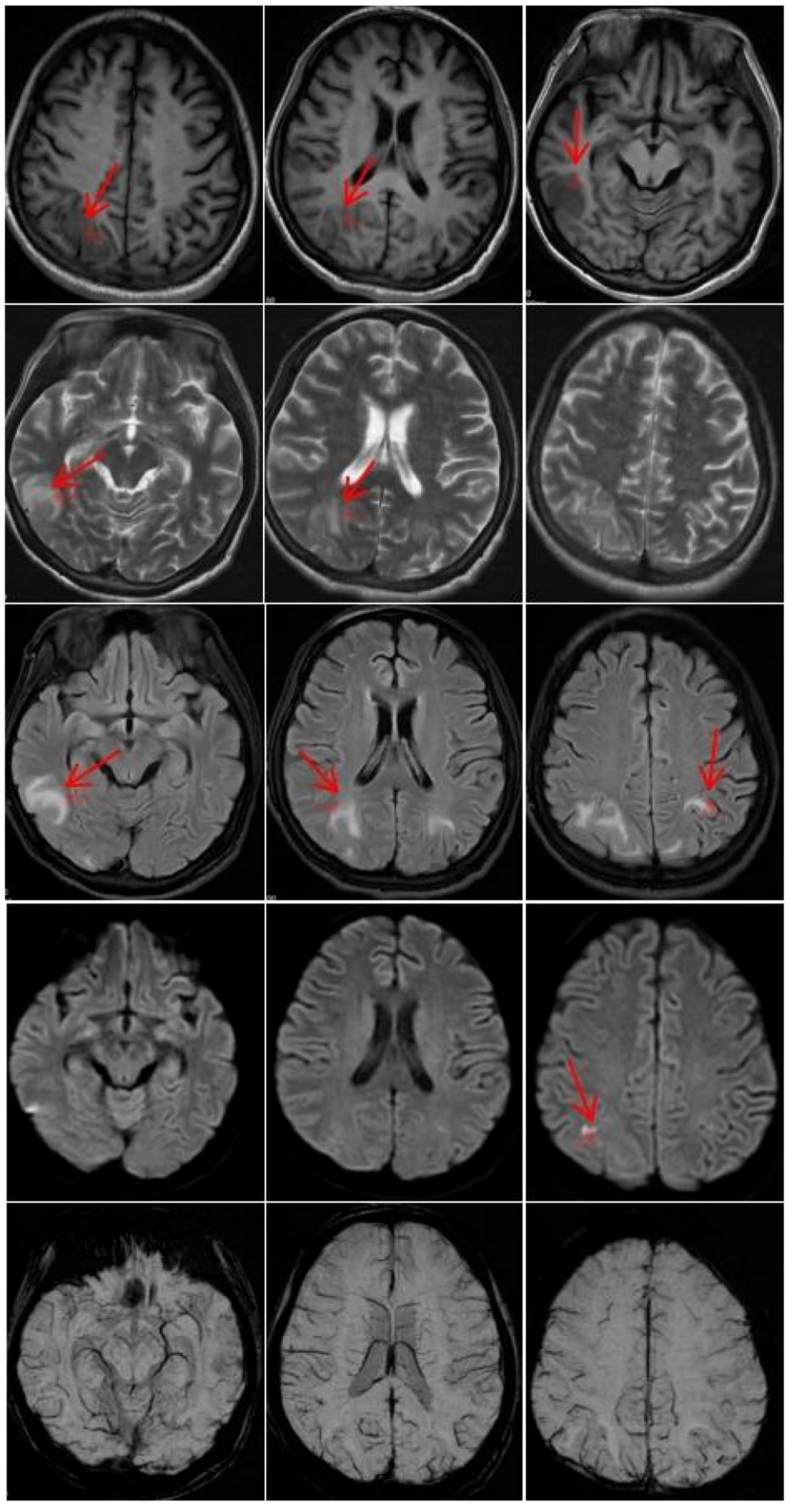
On April 11, 2022, cranial magnetic resonance imaging (MRI) revealed high-signal intensities in the bilateral parieto-occipital lobes and the right temporal lobe on T2-FLAIR sequences. Additionally, diffusion-weighted imaging (DWI) demonstrated localized areas of high signal. Susceptibility-weighted imaging (SWI) within the brain parenchyma did not detect any noticeable abnormal low-signal intensities. The neuroimaging features are highly suggestive of PRES syndrome diagnosis. The red arrows indicate lesion locations.

**Figure 2:**
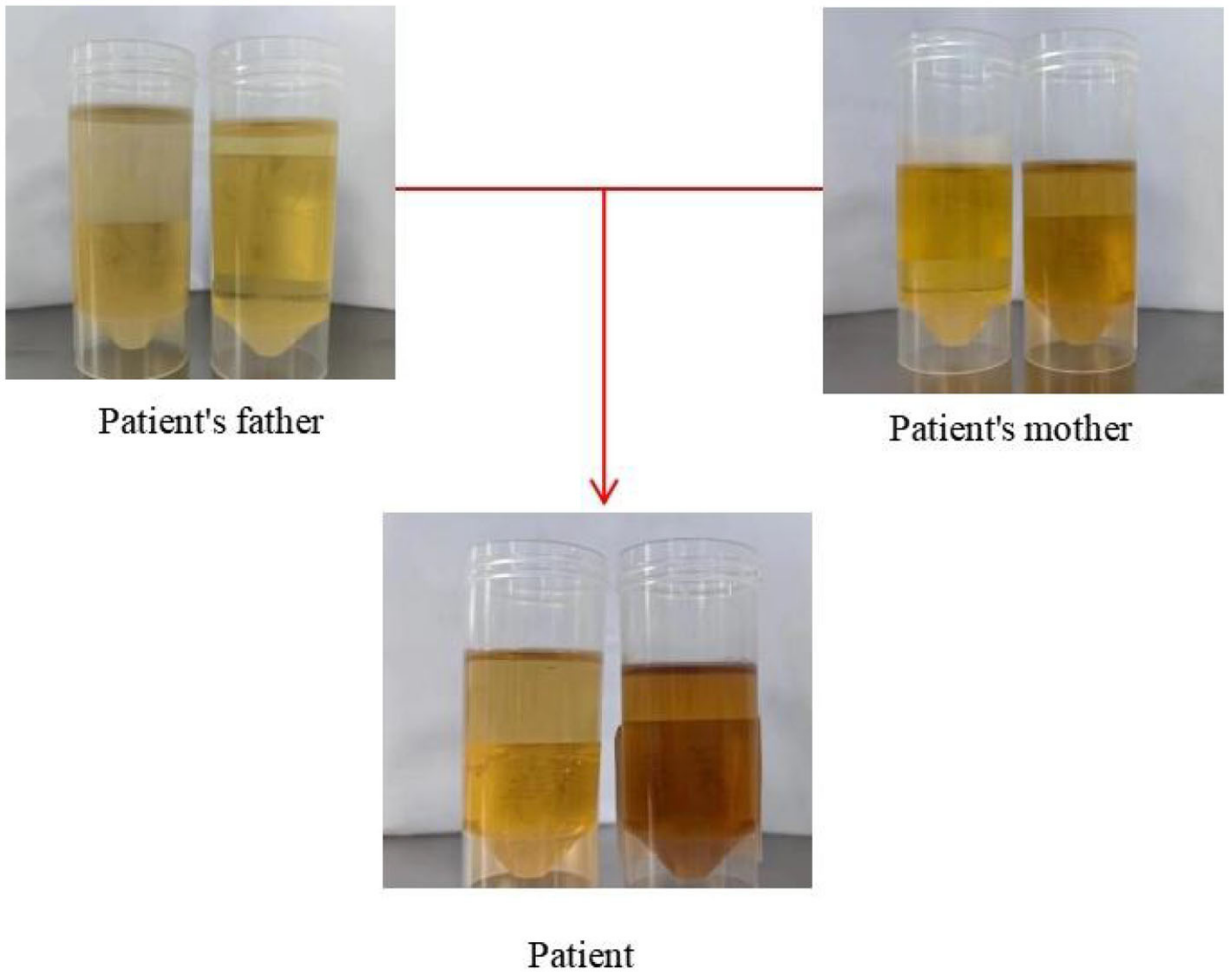
This figure displays the outcomes of urinary PBG solarization tests conducted on patient and her parents.

**Figure 3:**
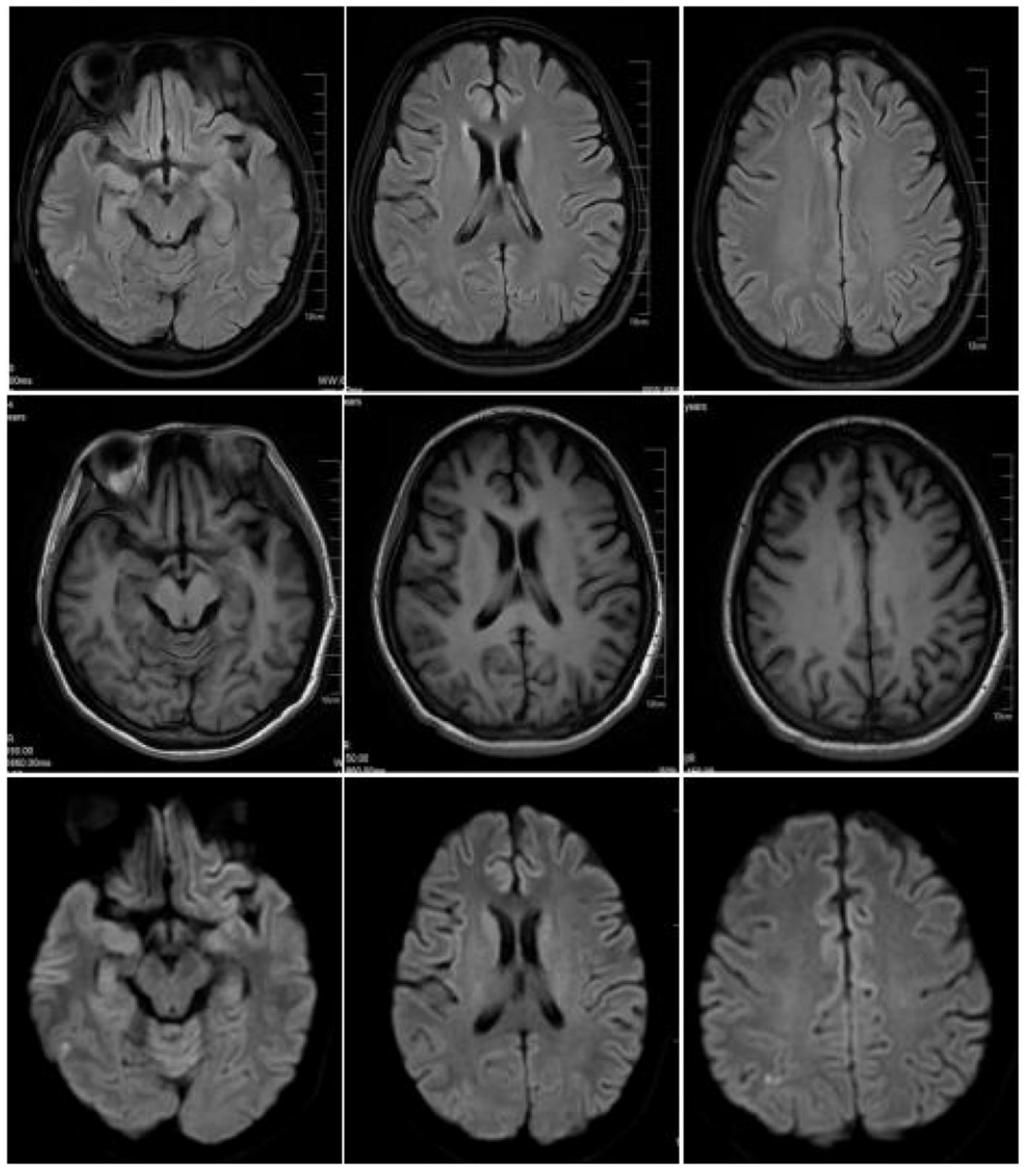
On April 28, 2023, cranial magnetic resonance imaging (MRI) revealed high-signal intensities in the bilateral parieto-occipital lobes and the right temporal lobe on T2-FLAIR sequences, while DWI sequences demonstrated localized areas of high signal. The extent of these signals has significantly decreased compared to the previous examination conducted on April 11, 2023.

On April 9, 2023, a 23-year-old woman was admitted to the hospital with a history of abnormal mood episodes for 4 years and weakness in the extremities for over 1 week. The patient had experienced intestinal obstruction in 2018, which was conservatively treated with satisfactory results, without any significant personal or family medical history. The patient began to exhibit mood abnormalities 4 years ago, manifesting as signs of depression and irritability. Over the years, the patient has frequently experienced abdominal and lumbar pain, leading her to engage in habitual jumping to alleviate these discomforts. These physical symptoms were accompanied by panic attacks, chest tightness, and difficulty sleeping. These symptoms often preceded menstruation and resolved spontaneously post-menstruation. Despite being treated with citalopram, olanzapine, and quetiapine at a hospital, the symptoms persisted. One month ago, the patient again became irritable and experienced insomnia, accompanied by unresponsiveness. Consequently, she was admitted to an external hospital, though the details of the consultation and treatment remain unknown. The symptoms did not improve significantly. One week prior to admission, the patient developed weakness in all four limbs, particularly the upper limbs, and progressively became bedridden. She also experienced dysphagia, shortness of breath, urination difficulties, and mental depression. Physical examination revealed moderate growth, malnutrition, a passive position, emaciation, and cyanosis. The patient had a urinary catheter and gastric tube in place. The remaining examination findings were unremarkable. The neurological examination showed that the patient was unconscious, non-verbal, and could only open her eyes in response to stabbing pain. The bilateral pupils were equal in size and round, with a diameter of approximately 4.0 mm, and the direct and indirect light reflexes were sensitive. The muscle strength of the limbs was weak, and there was a slight avoidance response to stabbing pain. Additionally, the patient exhibited decreased muscle tone in the extremities, absent tendon reflexes in the extremities, absent bilateral baroreflexes, neck tenderness, and kyphosis. Blood gas analysis indicated an oxygen partial pressure of 27 mmHg and carbon dioxide partial pressure of 68 mmHg, suggestive of type II respiratory failure. Despite being admitted to the hospital and receiving cardiac monitoring, high-concentration oxygen therapy, and sputum suction, the patient's SpO2 remained below 70%. As a result, she was intubated and ventilated with ventilator-assisted ventilation. Laboratory test results revealed a significant elevation in the white blood cell count at 24.52^*^109/L, a significantly reduced sodium ion concentration at 115.5 mmol/L, and an elevated calcitoninogen level at 0.530↑ng/mL. BNP, troponin I, coagulation routine, hepatic function, renal function, thyroid function, ANCA, and rheumatoid immune series did not exhibit any significant abnormalities. The results of infection markers, aspergillus serology test, and fungal D-glucan were within normal limits. On April 11, 2023, cranial magnetic resonance imaging (MRI) revealed high signal intensity in the bilateral parieto-occipital lobe and right temporal lobe in T2-FLAIR, local high signal intensity in the diffusion-weighted imaging (DWI) sequence, and a slightly low signal intensity in the corresponding ADC. No obvious abnormality of low signal intensity was observed in the brain parenchyma on the susceptibility-weighted imaging (SWI). Based on these imaging findings, reversible posterior white matter encephalopathy was considered (see Figure 1). Electromyography showed peripheral nerve damage in the upper and lower extremities involving both motor and sensory fibers, as well as axonal damage. The cell count in the cerebrospinal fluid was normal, while the protein concentration was elevated; ganglioside antibodies were negative in both blood and cerebrospinal fluid. Guillain-Barré syndrome (GBS) was suspected due to the acute onset and rapid progression of spinal and cranial nerve damage in the extremities, along with the presence of albuminocytological dissociation in the cerebrospinal fluid. Therefore, we administered human immunoglobulin (0.4 g/kg.d) for 5 days, methylcobalamin and lipoic acid to nourish the nerves, antibiotics to combat infection, and 3% hypertonic saline to correct hyponatremia, among other treatments. However, the patient's limb weakness did not show any significant improvement, and she still had difficulty with extrication and quadriplegia. Nine days after admission, the patient in the ICU experienced repeated loss of consciousness, failure to call out, and limb twitching. These symptoms could be terminated after administering sedative diazepam. Seizures were considered, so levetiracetam and sodium phenobarbital were added for antiepileptic purposes. Electroencephalogram showed a slowing of the background rhythm. At that time, we carefully considered the possibility of a misdiagnosis and further summarized the patient's condition. The patient had a history of intestinal obstruction and subsequently developed intermittent psychiatric abnormalities and abdominal pain related to the menstrual cycle. Recently, the patient had experienced acutely worsening peripheral neurological symptoms, which were consistent with the classic triad of acute intermittent porphyria. We observed that the patient's urine darkened to a dark brown color when exposed to sunlight (see Figure 2). Additionally, we collected urine samples from the patient's parents and found that the father's urine did not significantly change color after exposure to sunlight, while the mother's urine exhibited a mild darkening. Further genetic analysis of the patient's family line revealed that the patient carried a heterozygous missense variant in the *HMBS* gene (c.518G>A:p.R173Q), which was inherited from the patient's mother and was not present in the patient's father or younger brother. Ultimately, a definitive diagnosis of acute intermittent porphyria was made. Subsequently, we adjusted the patient's treatment regimen to include progesterone injections of 20 mg once daily (QD) intramuscularly to delay menstruation, dextrose injections of 150 g twice daily (BID) intranasally, and discontinued the use of aggravating medications such as sodium phenobarbital. On April 28, 2023, the patient's repeat cranial brain MRI revealed a significant decrease in the extent of the abnormal signal in the bilateral parietal-occipital lobes and right temporal lobe compared to the previous scan (see Figure 3). Despite being hospitalized for 32 days, the patient was still unable to be discharged, and her family requested an automatic discharge. At the time of discharge, she was coherent but mentally impaired, with normal comprehension and attention span. Her bilateral pupils were equal in size and round, measuring approximately 3 mm in diameter, and light reflexes were present. Both eyes were incompletely closed, and her bilateral frontal lines and nasolabial folds were symmetrical. The muscle strength of both upper limbs was grade 0, the distal muscle strength of both lower limbs was grade 1, and the muscle tone of all limbs was reduced. Tendon reflexes of the extremities were not elicited, and Babinski's sign was not present on both sides. The patient exhibited no signs of meningeal irritation, and the remaining portions of the examination could not be coordinated. On follow-up 10 days after discharge, the patient tolerated being weaned off the ventilator but still had weakness in all four limbs. Three months after discharge, the patient was on an intermittent high-sugar diet, and her limbs had significantly improved in strength, allowing her to stand on her own. Although she could breathe on her own, she still exhibited emotional irritability.

## Discussion

Porphyrias are a group of diseases resulting from enzyme deficiencies in the heme synthesis pathway, which leads to increased production of porphyrins and their precursors, δ-aminolevulinic acid (ALA) and porphobilinogen (PBG) (1). Heme, an iron porphyrin compound, serves as the cofactor for numerous crucial proteins in the blood, including hemoglobin, myoglobin, cytochromes, and peroxidase (6). Hemoglobin biosynthesis involves eight steps, and enzyme impairments at any of these steps can cause porphyrin dysregulation, resulting in the development of distinct porphyria subtypes. Based on the site of porphyrin production, they are classified as either hepatic or erythrocytic porphyrins (2). Acute intermittent porphyria (AIP) is the most prevalent form of acute hepatic porphyria (AHP) and is characterized by the accumulation of toxic neurological substances, such as porphyrins and their precursors, due to the absence of the third crucial enzyme in heme synthesis. Acute attacks manifest as the classic triad of abdominal pain, neurological symptoms, and psychiatric abnormalities (5). An analysis of the REBRAPPO study, which examines Brazilian patients suffering from porphyria, reveals that among 148 individuals diagnosed with AHP, the most common initial clinical manifestations were abdominal pain in 77 patients (52.0%) and acute muscle weakness in 23 patients (15.5%) (7). Similarly, a prospective, multinational observational study of patients with recurrent AHP found that 92% of patients experienced abdominal pain, while 91% presented with a change in urine color. The most common psychiatric symptoms were fatigue, anxiety, and sleep disturbances (8). However, a recent statistical analysis of AHP cases between 2012 and 2018 showed that only 32% of patients presented with the “classic triad” of symptoms during an attack. Additionally, 42% of patients exhibited two of these symptoms, while 55% reported dark-colored urine. Therefore, timely diagnosis of AIP remains a challenge for clinicians. Early diagnosis and effective treatment of AIP can significantly reduce the medical burden on both the patient's family and society, and improve prognosis (9). This case involves a young woman who was severely disabled by AIP for about 5 years, from the initial intestinal obstruction to the appearance of neurologic symptoms such as limb weakness and respiratory muscle weakness. During this time, she visited several hospitals. This patient presented with “classic triad” of AIP symptoms, including abdominal pain resulting from intestinal obstruction, neurological deficits manifesting as muscular weakness, and psychological anomalies in the form of sleep disturbances and alterations in mood. Due to the rarity of AIP and its non-specific symptoms, it is crucial that physicians across various specialties, including gastroenterology, neurology, psychiatry, and endocrinology, are aware of this disease to prevent potentially serious consequences of delayed diagnosis.

AIP is an autosomal dominant disorder caused by mutations in the *HMBS* gene, which encodes porphobilinogen deaminase (PBG deaminase). DNA sequencing of genes involved in heme biosynthesis has made it possible to pinpoint disease-causing mutations in an almost comprehensive manner for patients suffering from porphyria. However, it is noteworthy that significant genetic variations still remain elusive in a minority of patients exhibiting clinical and biochemical manifestations. The rapid development of next-generation sequencing (NGS) technologies could lead to an increase in the detection of missing genetic variants (10). DNA sequencing and analysis of *HMBS* genes to detect these mutations represent the gold standard for diagnosing AIP. Genetic diagnosis not only confirms the presence of AIP in individuals but also predicts disease severity and treatment responsiveness. The severity of clinical symptoms and disease progression in AIP appears to be mutation-specific. For instance, patients harboring p.W198X, c.1073delA, and p.R26C variants tend to exhibit more severe symptoms, while those with p.R167W, p.R225G, and c.G33T variants typically have a more indolent disease course with milder manifestations (11). In the present case report, we describe a patient with an *HMBS* gene mutation located at p.R173Q. Although the relationship between this specific mutation and disease severity remains unclear, our patient developed severe neurological damage and rapidly worsening symptoms of myasthenia gravis, suggesting a poor prognosis. This case underscores the importance of early genetic diagnosis in AIP to facilitate timely intervention and potentially alter disease outcomes.

AIP is not only an autosomal dominant disorder but also a monogenic one. Patients with AIP can retain up to 50% of enzyme activity when the mutated *HMBS* gene is inherited from one of their parents, a level sufficient for normal hemoglobin synthesis. As a result, most patients are asymptomatic carriers (12). AIP has been reported worldwide, with its distribution varying across regions and populations. *HMBS* gene variants have a low frequency in the general population but are more common in AIP families. Analysis of genome/exome sequencing data from 45,955 Caucasians revealed a prevalence of AIP of <1%, suggesting that acquired factors such as drugs, alcohol, hormones, starvation, and stress play a crucial role in the acute onset of the disease. The disease occurs in all races, with Europeans having a rate of *HMBS* gene mutations of approximately 1 in 1700. In contrast, the frequency of mutations in Caucasians may be as high as 6 in 1000. Studies conducted in 11 European countries found an annual incidence of symptomatic AIP of 0.13 per million. Due to the founder effect, the prevalence is significantly higher in the Nordic region, with rates of 0.51 and 0.22 per million reported in Sweden and Switzerland, respectively (13, 14). The *HMBS* variant rate in the Chinese general population is estimated to be 1 in 1765. Although the outlier rate is low, the large population size in China results in a considerable number of patients with symptomatic AIP. A retrospective study conducted on the general population of Hebei Province, China, between 2011 and 2020 showed an annual incidence of symptomatic AIP of approximately 0.03-0.08 per million. Given that the population of Hebei Province exceeds 70 million, it is estimated that there are around 400 patients with symptomatic AIP (15). Upon comparing and analyzing the data from the aforementioned studies, it becomes apparent that the incidence of symptomatic AIP in China is significantly lower than that in Europe. This difference can be attributed to the early recognition of porphyria in Europe and the establishment of a robust diagnostic and management system for AIP patients, along with enhanced family screening and preventive counseling (16). Furthermore, while there is no gender difference in carriers of the *HMBS* gene variant, the majority of studies indicate a higher proportion of acute exacerbations in female patients with AIP compared to males, suggesting a close association between female hormones and the development of AIP (17).

Most patients experience symptom onset between the ages of 20 and 40, with a smaller proportion experiencing symptoms during the prepubertal and postmenopausal periods. AIP typically manifests as acute, intermittent episodes, and its clinical presentations are complex and varied, making it challenging to differentiate from other common disorders solely through clinical assessment. AIP can affect the autonomic nervous system, the peripheral nervous system, and the central nervous system. Abdominal pain is the most common and earliest autonomic symptom of an attack, occurring in over 90% of patients (18). This pain is typically intermittent colic without significant pressure and is often accompanied by nausea, vomiting, and constipation. Patients' episodes of abdominal pain are acute and severe, and are frequently misdiagnosed as acute abdominal diseases such as intestinal obstruction, appendicitis, pancreatitis, among others. Some patients undergo unnecessary surgical treatments due to recurrent episodes that do not allow for a clear etiology. Additionally, these gastrointestinal symptoms lead to decreased appetite and reduced energy intake, resulting in negative energy balance in patients, which further exacerbates attacks (19). A large-scale, population-based survey study conducted in the United States in 2020 revealed that approximately one-fourth of patients with a history of abdominal pain experienced symptoms similar to those of acute hepatic porphyria (20). Although the incidence of AIP is low, it remains a crucial differential diagnosis in patients presenting with recurrent episodes of severe abdominal pain. Autonomic neuropathy can also induce other symptoms, such as hypertension, tachycardia, diarrhea, postural hypotension, and abnormal sweating (21). Neuropsychiatric symptoms in AIP patients manifest in various forms, lacking specificity and occurring at any given time. The typical manifestation of peripheral neuropathy is acute motor nerve axonal damage, initially affecting proximal muscles and upper limbs, gradually progressing to lower limbs and distal extremities, primarily manifesting as muscle weakness. In severe cases, motor polyneuropathy can result in quadriplegia and respiratory failure (22, 23). In some patients, sensory nerves are involved, resulting in a “glove-and-stocking” distribution of sensory deficits, which may manifest as neuralgia, hyperalgesia, or numbness, with loss of nociceptive sensation being less common (24). Epilepsy occurs in up to 20% of AIP patients, typically manifesting as complex partial seizures, myoclonic jerks, and tonic-clonic seizures (25). Additionally, other symptoms of acute AIP onset may induce seizures, such as hyponatremia and post-reversible encephalopathy syndrome (PRES) (26). PRES is a reversible subcortical vasogenic cerebral edema, characterized by symptoms such as headache, seizures, encephalopathy, and visual disturbances. AIP is a rare cause of this condition (27). Recent studies examining the clinical features of AIP patients with PRES have revealed that the mean age of onset for PRES is between 24–44 years, with a significant predominance of women over men. Seizures were present in 85%-95% of PRES patients (28, 29). Hyponatremia is the most commonly observed laboratory abnormality during acute seizures of AIP, which may be associated with the syndrome of inappropriate antidiuretic hormone secretion (SIADH) (30). During an acute seizure of AIP, damage to the hypothalamus resulting from a large accumulation of porphyrin precursors and abnormal hemoglobin activity leads to increased vasopressor secretion and substantial fluid retention, ultimately causing dilutional hyponatremia (31). Frequent and debilitating chronic symptoms affect over 65% of patients with AHP, with a substantial proportion of these symptoms being linked to neurological and psychiatric conditions. Such manifestations include severe chronic pain (whether abdominal, neuropathic, or diffuse myalgia), anxiety and mood disorders, fatigue, sleep disturbances (notably insomnia), and muscle weakness that impede daily routines. These symptoms, in turn, can serve as precipitants for acute episodes of AIP, leading to exacerbation of the condition (32, 33). In addition to affecting the nervous system, abnormalities in porphyrin metabolism can also cause disorders of the endocrine and metabolic systems. The prevalence of insulin resistance and impaired glucose metabolism is higher among AIP patients compared to the general population (34). However, there is a scarcity of literature on the impact of porphyrin metabolism on the endocrine system, and further investigation is warranted to elucidate its pathogenesis. This case involved abdominal pain presenting 5 years ago. At that time, due to atypical clinical symptoms, the clinician treated it as the most common abdominal disease, intestinal obstruction, which had a fair effect. However, later on, intermittent mood abnormalities accompanied by abdominal pain emerged. Upon presentation to our hospital, the patient exhibited weakness in the extremities, respiratory failure, reversible posterior leukoencephalopathy imaging findings, hyponatremia, and seizures. The patient had a history of acute intermittent seizures for ~5 years, ultimately leading to quadriplegia and a poor prognosis. This case highlights that AIP is prone to misdiagnosis and delayed diagnosis, which can significantly impact the patient's prognosis.

The exact mechanism behind the pathogenesis of AIP remains elusive, with studies revealing significant elevations in plasma levels of cytokines, chemokines, growth factors, and other inflammatory markers among AIP patients. This suggests a potential association between AIP and low-grade inflammation. Perforin 3 (PTX3), an inflammatory mediator produced by immune cells, emerges as a key player in this low-grade inflammation and serves as a marker of AIP disease activity (35). The dissemination of ALA from the liver to other body parts contributes significantly to the neurological manifestations observed in AIP. High concentrations of ALA exhibit neurotoxicity and bear structural similarities to γ-aminobutyric acid (GABA). Consequently, ALA acts as a partial agonist or antagonist of GABA at GABA receptors, leading to neuronal cell damage and apoptosis. Additionally, high levels of ALA impair glutamate uptake by augmenting oxidative stress. The occurrence of confusion, epilepsy, and psychiatric abnormalities during acute seizures in AIP may be linked to these underlying mechanisms (36). Another perspective suggests that prolonged heme deficiency in AIP patients impairs neuronal axonal transport. During acute seizures of AIP, severe hepatic heme deficiency disrupts tryptophan metabolism, and excessive tryptophan hinders neurotransmission, resulting in neurological dysfunction. To replenish the heme pool, the liver exerts negative regulation on the first rate-limiting enzyme in the heme synthesis pathway, 5-aminolevulinic acid synthetase-1 (ALAS1), which generates substantial amounts of ALA and partially converts it to PBG. Patients with AIP lack the enzyme PBG deaminase, ultimately leading to the accumulation of porphyrin precursors, ALA, and PBG (18).

Cranial MR imaging plays a crucial role in the diagnosis of AIP. Hematoporphyrinopathies involve lesions located in the cortex or subcortical white matter of each lobe of the brain, primarily affecting white matter and often presenting symmetrically. In MRI, these lesions are characterized by long T1 and long T2 signals, with some lesions fused, exhibiting low signals in DWI, high signals in ADC, and high signals in FLAIR. Reversibility was observed in some lesions, resembling the distribution and characteristics of lesions seen in PRES (37). The pathogenic basis of AIP-associated PRES remains elusive. Previous studies have posited that hypertension triggers a loss of control in autoregulatory mechanisms, leading to secondary hyperperfusion during an acute episode of AIP. This results in the rupture of the blood-brain barrier and leakage of fluid from the vascular lumen into the brain interstitium, ultimately causing vasogenic edema. Additionally, it has been suggested that the heme deficiency in the body during an acute attack of AIP decreases nitric oxide (NO) production, leading to an increase in blood pressure and cerebral vasoconstriction (38). During an acute attack of AIP, ischemic changes may occur, which can be reversible or irreversible. In the former case, reversible vasospasm is present without any organic lesions visible on cranial MRI. However, in the latter case, foci of softening can be observed on MRI imaging. Severe stenosis of cerebral vessels may even manifest as stroke-like symptoms (39). Moreover, AIP presents with some nonspecific electrophysiologic manifestations, including increased slow waves or epileptic discharges on electroencephalogram, and neurogenic damage and motor neuron axonal injury rather than demyelinating neuropathy on electromyography (40). Cerebrospinal fluid examination does not reveal typical protein separation. Biochemical tests, such as the urine PBG sunlight test, are crucial in diagnosing AIP. In patients with AIP, the test results are positive, as fresh urine exposed to sunlight for several hours turns reddish-brown or burgundy due to the conversion of colorless PGB in urine to colored porphyrin compounds. Collecting random urine samples during an acute attack and qualitatively testing for urinary PBG allows for rapid screening of porphyrias (41). The analysis of urinary PBG, ALA, and porphyrin levels in fecal and blood samples obtained from patients experiencing an acute attack of AIP serves as a valuable diagnostic tool. However, solely relying on this approach may not be sufficient. Genetic testing can properly identify pathogenic or suspected variants in PPOX, CPOX, ALAD or HMBS genes associated with Acute Hepatic Porphyria (42).

AIP is a complex multisystem disease with diverse clinical manifestations, making prompt diagnosis a challenging task. When AIP presents as acute abdominal pain, it is crucial to differentiate it from various acute abdominal conditions to exclude organic abdominal disease, such as intestinal obstruction and inflammatory diseases. Testing urine PBG levels can aid in the differential diagnosis. Guillain-Barré syndrome typically does not manifest with psychiatric symptoms, and porphyrins and their metabolites are typically within normal limits. Lead poisoning can result in elevated porphyrin or ALA levels, which can mimic the clinical presentation of AIP. Therefore, clinicians must focus on differentiating lead poisoning when diagnosing acute porphyrias. The clinical manifestations of lead poisoning depend on blood lead levels, with high levels leading to anemia, constipation, abdominal pain, peripheral neuropathy, and even impaired consciousness. Patients with lead poisoning typically have a clear history of lead exposure and significantly elevated blood and urine lead levels (43). An elderly woman with chronic, long-term gastrointestinal symptoms including abdominal pain and weight loss, as well as chronic neurologic symptoms of memory impairment, has recently been reported. This patient exhibited elevated urinary porphyrins and was initially diagnosed with acute porphyria; however, given the patient's age, it was imperative to consider alternative diagnoses. Subsequently, the patient was found to have significantly elevated blood lead levels, ultimately leading to a final diagnosis of lead poisoning (suspected ayurvedic use) (44). This case underscores the complexity of diagnosing porphyria and emphasizes the need for clinicians to enhance their understanding of AIP to prevent delayed misdiagnosis.

The principles of treatment for AIP encompass the elimination of the causative agent, management of the acute exacerbation phase, treatment of recurrent episodes, and prevention and treatment of complications. AIP is caused by a variant of the HMBS gene, but the manifestation of the disease phenotype requires additional genetic or acquired factors. Acute attacks of AIP can be precipitated by menstruation, pregnancy, infection, stress, inadequate caloric intake, and certain medications such as barbiturates, antiepileptic drugs, sulfonamide antibiotics, and steroid hormones like progesterone (5). Prevention of acute AIP attacks involves identifying triggers and avoiding exposure to them, including reducing or avoiding smoking and alcohol consumption, maintaining a healthy diet with adequate intake of carbohydrates, proteins, and fats to minimize porphyrin precursor excretion, and avoiding dieting in patients with porphyria (45). Additionally, patients should avoid oral porphyrin-derived medications that induce cytochrome P450. Those with AIP associated with epilepsy should exercise caution in selecting antiepileptic drugs; for instance, phenytoin sodium and carbamazepine induce CYP450, whereas gabapentin and levetiracetam are more suitable alternatives (46). Patients with AIP related to the menstrual cycle may consider taking oral contraceptives to prevent acute attacks. The preferred treatment for acute-phase episodes of AIP is intravenous hemin, which effectively inhibits ALAS1, reducing the accumulation of heme precursors and their byproducts, and rapidly decreasing plasma and urinary levels of PBG and ALA. Due to the instability of heme in water, it should be reconstituted with 25% human albumin. The optimal route of administration is via a central venous catheter, with a standard dosing regimen of 3–4 mg/kg/day for 4 days. If complete remission is not achieved within this period, the duration of treatment may be extended. Hemin is a safe option to use during pregnancy and should be administered as soon as possible following an acute episode of AIP. If hemin is unavailable, carbohydrate-loading therapy can be administered orally or intravenously with glucose. Good therapeutic outcomes can also be achieved effectively with glucose alone in patients with AHP (47). Glucose stimulates insulin release by reducing peroxisome proliferator receptor gamma coactivator 1-α (PGC1-α), which mediates hepatic ALAS1 downregulation and impairs ALA synthesis. In the early stages of AIP episodes, when mild pain is present without severe clinical manifestations, glycogen loading therapy (300 g/day) can be utilized. It is crucial to note that large amounts of intravenous glucose may result in hemodilution, potentially exacerbating the risk of hyponatremia (48). Symptomatic supportive therapy is provided when other complications are present: opioid analgesics such as morphine and pethidine are relatively safe in acute episodes of abdominal pain; gabapentin and levetiracetam are recommended for patients with epileptic seizures; and for patients with severe hyponatremia, 3% hypertonic saline is administered via intravenous infusion (49). Emerging Therapies—Givosiran is a highly specific, double-stranded small interfering ribonucleic acid (siRNA) designed to bind and silence ALAS1 mRNA within hepatocytes. This action inhibits the synthesis of ALAS1, leading to a decrease in circulating δ-ALA and PBG levels among patients experiencing AHP and severe relapses. In the ENVISION Phase 3 trial, which was conducted in a randomized, double-blind, and multinational setting, givosiran demonstrated a significant reduction in the annual incidence of acute attacks, including those necessitating hospitalization, emergency medical interventions, or at-home intravenous hemoglobin administrations. Furthermore, patients with relapsing AIP who received Givosiran showed decreased reliance on hemoglobin infusions and pain medications compared to those given a placebo. The available evidence strongly indicates that Givosiran represents a pivotal new treatment modality for individuals with AHP and severe relapses (50). Liver transplantation remains the last effective resort for managing all symptoms in critically ill patients who have exhausted other treatment options. However, challenges such as donor shortage and long-term oral immunosuppression post-transplantation must be faced by patients (51). AIP is associated with a high incidence of chronic complications like hypertension, chronic kidney disease, and hepatocellular carcinoma. Therefore, patients with AIP require continual monitoring of liver and kidney function, along with regular screening for hepatocellular carcinoma (9). As hemoglobin preparations are unavailable in our hospital, we administered progesterone injections to delay menstruation and prevent acute attacks, supplemented by glucose-loading therapy. Eventually, the patient and her family opted for automatic discharge, and the patient was discharged from the hospital on an intermittent high-glucose diet, with significant improvement in limb muscle strength and spontaneous respiration.

## Conclusion

Acute intermittent porphyria is a rare and clinically demanding disease. Only a small fraction of patients manifest the “classic triad,” and there often exists a considerable lag between the advent of symptoms and the establishment of a diagnosis. Given its multi-systemic nature, it is imperative for clinicians across neurology, psychiatry, gastroenterology, and other fields to recognize this ailment, thus averting any delays in diagnosis and treatment. The presence of unexplained abdominal pain, accompanied by neurologic and psychiatric signs, should serve as a red flag for porphyria. If the urine darkens to a deep red or wine-colored hue upon exposure to sunlight and the urine PBG test returns positive, further genetic testing can be conducted to affirm the diagnosis and typing, thereby expediting the confirmation of hematoporphyria and the prompt initiation of appropriate treatment. We present a case exhibiting a myriad of typical clinical manifestations of AIP, in the hope of aiding clinicians in gaining a comprehensive understanding of the disease and facilitating timely and accurate diagnosis in patients presenting with analogous symptoms.

## Data availability statement

The original contributions presented in the study are included in the article/supplementary material, further inquiries can be directed to the corresponding author.

## Ethics statement

The studies involving humans were approved by Ethics Committee of the Shandong Provincial Qianfoshan Hospital. The studies were conducted in accordance with the local legislation and institutional requirements. The participants provided their written informed consent to participate in this study. Written informed consent was obtained from the individual(s) for the publication of any potentially identifiable images or data included in this article.

## Author contributions

JLin: Writing – original draft. JLiu: Writing – review & editing. AW: Writing – review & editing. ZS: Writing – review & editing.
